# Changes in the innate immune response to SARS-CoV-2 with advancing age in humans

**DOI:** 10.1186/s12979-024-00426-3

**Published:** 2024-03-21

**Authors:** Sudhanshu Agrawal, Michelle Thu Tran, Tara Sinta Kartika Jennings, Marlaine Maged Hosny Soliman, Sally Heo, Bobby Sasson, Farah Rahmatpanah, Anshu Agrawal

**Affiliations:** 1 Division of Basic and Clinical Immunology, Department of Medicine, University of California Irvine, Irvine, CA 92697 USA; 2 Department of Medicine, University of California Irvine, Irvine, CA 92697 USA; 3 Department of Pathology, University of California Irvine, Irvine, CA 92697 USA

**Keywords:** SARS-CoV-2; innate immunity, DCs, Monocytes, IL-29, RNA-seq, Aging, CD8 T cells

## Abstract

**Background:**

Advancing age is a major risk factor for respiratory viral infections. The infections are often prolonged and difficult to resolve resulting hospitalizations and mortality. The recent COVID-19 pandemic has highlighted this as elderly subjects have emerged as vulnerable populations that display increased susceptibility and severity to SARS-CoV-2. There is an urgent need to identify the probable mechanisms underlying this to protect against future outbreaks of such nature. Innate immunity is the first line of defense against viruses and its decline impacts downstream immune responses. This is because dendritic cells (DCs) and macrophages are key cellular elements of the innate immune system that can sense and respond to viruses by producing inflammatory mediators and priming CD4 and CD8 T-cell responses.

**Results:**

We investigated the changes in innate immune responses to SARS-CoV-2 as a function of age. Our results using human PBMCs from aged, middle-aged, and young subjects indicate that the activation of DCs and monocytes in response to SARS-CoV-2 is compromised with age. The impairment is most apparent in pDCs where both aged and middle-aged display reduced responses. The secretion of IL-29 that confers protection against respiratory viruses is also decreased in both aged and middle-aged subjects. In contrast, inflammatory mediators associated with severe COVID-19 including CXCL-8, TREM-1 are increased with age. This is also apparent in the gene expression data where pathways related host defense display an age dependent decrease with a concomitant increase in inflammatory pathways. Not only are the inflammatory pathways and mediators increased after stimulation with SARS-CoV-2 but also at homeostasis. In keeping with reduced DC activation, the induction of cytotoxic CD8 T cells is also impaired in aged subjects. However, the CD8 T cells from aged subjects display increased baseline activation in accordance with the enhanced baseline inflammation.

**Conclusions:**

Our results demonstrate a decline in protective anti-viral immune responses and increase in damaging inflammatory responses with age indicating that dysregulated innate immune responses play a significant role in the increased susceptibility of aged subjects to COVID-19. Furthermore, the dysregulation in immune responses develops early on as middle-aged demonstrate several of these changes.

**Graphical abstract:**

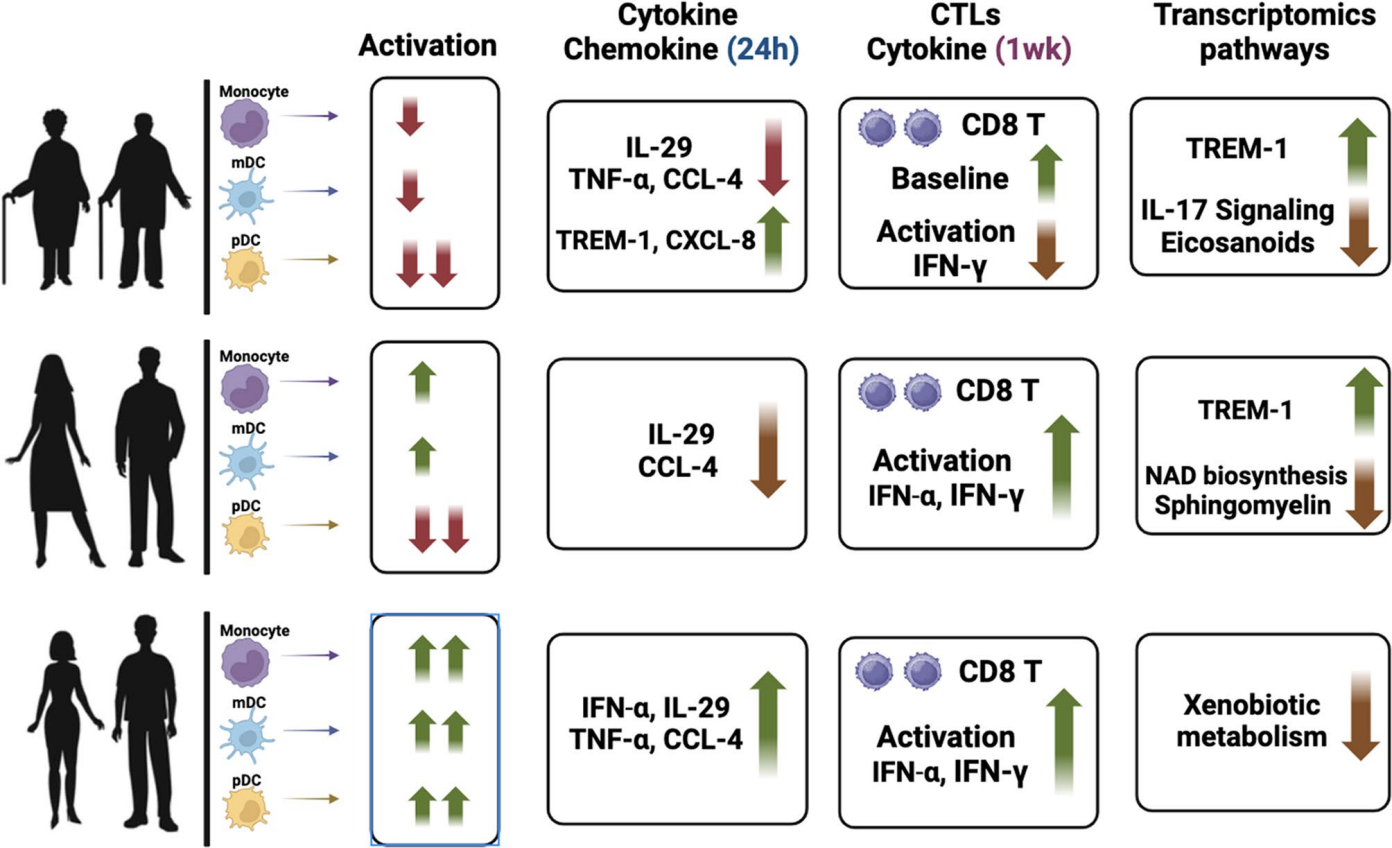

**Supplementary Information:**

The online version contains supplementary material available at 10.1186/s12979-024-00426-3.

## Background

The COVID-19 pandemic has affected millions of individuals worldwide [1, 2]. The majority of infected individuals have mild illness, and many may be asymptomatic. Those with serious illness develop severe respiratory complications associated with increased proinflammatory cytokines, including CXCL-10, CCL-2, and TNF-α, in the plasma [3, 4]. This so-called “cytokine storm” can initiate viral sepsis and inflammation-induced lung injury, which lead to other complications, including pneumonitis, acute respiratory distress syndrome (ARDS), respiratory failure, shock, and potentially death [3, 4]. Advanced age has emerged as a major risk factor. The number of hospitalizations and mortality increases substantially from age 50 years and more. In fact, the CDC reported that eight out of ten deaths due to COVID-19 were in individuals 50 years or older. Comorbidities including diabetes, hypertension, and heart disease add further to the risk [2, 5–8]. Though vaccination has significantly reduced the risk of COVID complications and hospitalization, according to CDC older adults still accounted for 63% of all COVID-related hospitalizations and approximately 90% of deaths in 2023 in the United States [9, 10]. Data from the COVID-19–Associated Hospitalization Surveillance Network (COVID-NET) also indicates that hospitalization rates were higher in subjects 65 years and older in 2023 [9]. Age-related dysregulated immunity is considered the primary culprit, although the mechanisms are not well understood.

Innate immunity is the first line of defense against viruses, and there is a scarcity of information about changes in innate immune functions in aged individuals who display increased susceptibility and severity to SARS-CoV-2. Dendritic cells (DCs) and macrophages are key cellular elements of the innate immune system that can sense and respond to viruses by producing inflammatory mediators and priming CD4 and CD8 T-cell responses [11]. DCs produce type I and III interferons (IFNs) and other cytokines that enhance the antiviral activity of other cells and prime antiviral cytotoxic CD8 T cells [12]. Emerging data indicate that CD8 T-cell function is dysregulated in patients with severe COVID-19 [13, 14]. CD8 T cells display reduced expression of CD107a, suggesting decreased degranulation and release of granzyme B. The production of IFN-γ and TNF-α is also impaired, and the expression of PD-1 is upregulated [15]. DCs prime naïve T cells, and changes in the function of DCs in vulnerable populations may be responsible for the reduced CD8 T-cell priming observed in severe patients. Data from our laboratory indicate that although the percentages of DC subsets in the circulation are comparable between aged subjects and young subjects [16], DCs from elderly subjects display enhanced secretion of proinflammatory cytokines, TNF-α, IL-6, etc., at baseline [17]. The increased inflammation at baseline impairs the response of DCs to infections, where they display reduced secretion of protective antiviral cytokines and type I and type III interferons and impaired priming of CD4 and CD8 T cells [18, 19]. We also found a reduction in antigen-presenting molecules in DCs from aged subjects [20]. The presence of diabetes, hypertension and heart disease further enhances the risk. Only a limited number of studies have examined DCs; however, in these subjects, DCs were also observed to display increased inflammation at baseline [21–23].

In contrast to DCs, alveolar macrophages maintain homeostasis in the airways and manifest an anti-inflammatory response to protect against excessive tissue damage induced during viral infections [12]. However, during infections, monocytes from circulation can infiltrate the lung and differentiate into inflammatory macrophages [24]. Studies indicate that there are increased proinflammatory monocyte-derived macrophages in the bronchoalveolar lavage (BAL) fluid of patients with severe COVID-19 [25, 26]. Our studies indicate an increase in the percentages of inflammatory monocytes (CD14^+^CD16^++^) in elderly individuals [27]. An increase in inflammatory monocytes is also observed in diabetics, where a higher expression of TNF-α and IL-8 was detected in monocytes [24, 28]. Similarly, an increase in inflammatory monocyte frequencies was also observed in hypertensive subjects [28, 29]. Given the pivotal roles of monocytes and DCs in host defense against viral infections, it is important to determine whether SARS-CoV-2 can interfere with the function of these two critical immune cells, thereby influencing the outcome of the infection. The inflammatory phenotype of DCs and monocytes in the elderly may play a role in the exaggerated cytokine response.

The primary objective of this study is to determine the response of DCs and monocytes to SARS-CoV-2 as a function of age. We used PBMCs from aged, middle-aged, and young subjects to determine the response to SARS-CoV-2. Improved insight into the underlying mechanisms that render older individuals more susceptible to developing severe disease may be helpful in the development of new candidate strategies for therapeutic intervention not only for COVID-19 but also for other respiratory infections.

## Methods

### Blood donors

Peripheral blood samples were obtained from volunteers (21–90 years) with the help of the Institute for Clinical and Translational Immunology (ICTS), UC Irvine and internal medicine clinic at UC Irvine. Individuals with infections, medications that modify the immune response and cancer are excluded. Subjects between 21 and 63 years were primarily healthy while older subjects (65 years and older) had certain comorbidities mentioned in Table 1. The Institutional Review Board (IRB) of the University of California (Irvine, CA, USA) approved the protocol. Written, informed consent was obtained from the subjects. Table 1 describes the cohort. Blood was collected in BD Vacutainer tubes with sodium heparin. Fresh PBMCs were used for the experiment.

**Table 1:** Description of cohort of subjects used in the study

No of subjects	Sex	Age range (Years)	Disease	Vaccinated
10 (Young)	Female	21–40	none	yes
10 (Young)	Male	21–40	none	yes
12 (Middle-aged)	Female	41–63	none	yes
8(Middle-aged)	Male	41–63	none	yes
2 (Aged)	female	68–90	none	yes
5 (Aged)	female	68–90	HTN + Hyper Lipidemia	yes
2 (Aged)	female	68–90	HTN + DM2	yes
3 (Aged)	female	68–90	HTN	yes
6 (Aged)	Male	68–85	HTN and DM2	yes
2 (Aged)	Male	68–85	none	yes

*N* = 20/each age group

HTN-hypertension; DM2- Type 2 Diabetes Mellitus. All older subjects received Moderna or Pfizer vaccine or a combination of both. The information of vaccine brand for young and middle-aged subjects was not collected.

### Antibodies and reagents

The antibodies used for staining are listed in supplementary Table 11 (ST4) in additional files.

Irradiated SARS-CoV-2 virus isolate USA-WA1/2020 was obtained from Bei Resources (NIAID). As per the BEI resources, gamma irradiation was performed using 5 × 10^6^ RADs on dry ice. The inactivated viruses are biosafety level 1.

### Viral stimulation

Peripheral blood mononuclear cells (PBMCs) were isolated from the blood of volunteers by density gradient centrifugation using lymphocyte separation media (Mediatech, Fisher Scientific, USA). Fresh 5 × 10^6^ PBMCs/2 ml were stimulated with 10 µg of irradiated SARS-CoV-2 virus in RPMI 1640 containing 10% fetal bovine serum (Gibco, Thermo Fisher Scientific, USA) as described previously [30]. After 24 h, half of the cells and supernatants were collected. The collected cells were divided into two halves. Half were stored in a DNA/RNA shield for RNA extraction. The remaining half was stained for activation of DCs and monocytes using specific antibodies as described. Supernatants were stored at -70 °C for quantitation of cytokines and chemokines. The remaining cells were cultured for another six days. Subsequently, the cells were collected and stained for cytotoxic T lymphocytes. Supernatants were stored at -70 °C for quantitation of adaptive immune cytokines. Summary methodology figure is included as supplementary figure S1.

### Immuno-phenotyping (flow cytometry)

PBMCs collected after stimulation for 24 h were stained with fixable viability stain 510 (BD Biosciences) for live/dead cell exclusion as per the manufacturer’s instructions. The cells were then washed, and surface stained for DCs and monocytes using specific antibodies for 30 min at RT in the dark. Subsequently, the cells were washed and fixed using 2% PFA. The required FMO and isotype controls were prepared in the same way. Cells were acquired by BD FACS Celesta (Becton-Dickenson, San Jose, CA) equipped with a BVR laser. Forward and side scatters and singlets were used to gate and exclude cellular debris (Supplementary figure S2). Thirty thousand cells were acquired/sample. Analysis was performed using FlowJo software (Ashland, OR). DCs were identified by the following phenotypes: mDCs, lin^−^/HLA-DR^+^/CD11c^+^; pDCs, lin^−^/HLA-DR^+^/CD123^+^; and monocytes, CD14^+^/HLA-DR^+^. The expression of CD86 was determined in these gated populations.

For cytotoxic CD8 T-cell staining, the cells collected on day 7 were stained with fixable viability stain 510 for live/dead cell exclusion. After washing, the cells were surface stained with CD8 antibody for 30 min. The cells were then fixed and permeabilized with fix/perm buffer (BD Biosciences) and stained with Granzyme B. Appropriate FMO and isotype controls were used for all staining (supplementary figure S3). Acquisition and analysis were performed as described above.

### Multiplex cytokine/chemokine assay

Culture supernatants collected 24 h and one week post stimulation were assayed using Multiplex kits (Thermo Fisher Scientific, USA). The specific kit used for 24 h culture supernatant contained the following analytes: IL-10, IL-6, IL-8, IL-29, CCL-2, TNF-α, IFN-α, CXCL-10, CCL-19, IL-18, TREM-1, IL-1Ra, GM-CSF, IL-15, and PDGF-BB. The kit for seven-day supernatants contained the analytes IL-29, TNF-α, IFN-α, IL-6, IFN-γ, IL-17 A, IL-1β, and granzyme B. The procedure was performed according to the manufacturer’s protocol. Briefly, the supernatant was mixed with premixed beads overnight, and after incubation with detection antibodies and streptavidin-PE for 1 h each, the plate was run on Magpix to identify specific cytokines.

### RNA extraction, sequencing, and analysis

RNA was extracted from the cells stored in DNA/RNA shield using a Quick DNA/RNA miniprep kit (Zymo Research, Irvine, CA) following the manufacturer’s protocol. The quality assessment, library preparation and sequencing were performed by UCI Genomics Research and Technology Hub, GRTH). Transcriptome analysis was performed as described previously [31–34]. Millions of reads for each of the samples were QC analyzed based on the quality score distribution. Processed reads were mapped to the reference genome (hg38) using Strand NGS v1.3 (data analysis package). Deduplication was performed to remove duplicate reads generated by PCR amplification bias during library construction. Deduplicated sequencing reads were quantitated for expression levels. The raw counts were normalized using the DESeq normalization method. Normalized counts are log transformed and baselined based on the mean expression levels of all samples (Strand NGS—RNA seq analysis). Pooled analysis was performed using the Audic-Claverie Test (AC) test. The Benjamini–Hochberg correction was applied to account for multiple testing, and a corrected *p* value of 0.05 was used as the threshold for the detection of differentially expressed genes.

Pathway analysis, including comparative pathway analysis, was performed using Ingenuity pathway analysis software (Qiagen, Germany). Values equal to or above 1.3 are considered significant.

### Statistical analyses

Statistical analysis was performed using GraphPad Prism version 9 (GraphPad Inc., San Diego, CA, USA). Parametric Paired t test with 95% confidence interval was used for comparisons between two groups. One-way ANOVA followed by Tukey’s multiple comparison test was used for the analysis of two or more groups. For ANOVA also the confidence interval was 95%. A *P* value of < 0·05 was considered statistically significant. Statistical considerations for each figure are provided in the figure legend.

## Results

### Activation of DCs and monocytes by IRR SARS-CoV-2 in PBMCs as a function of age

Immune responses and respiratory lung function in aged subjects are not as efficient as those in the young population. This is evident from the higher morbidity to COVID-19 observed in elderly individuals. In the present study, we compared the immune responses to SARS-CoV-2 virus in young (22–40 years), middle-aged (41–63 years) and aged (68–90 years) subjects.

**Fig. 1:**
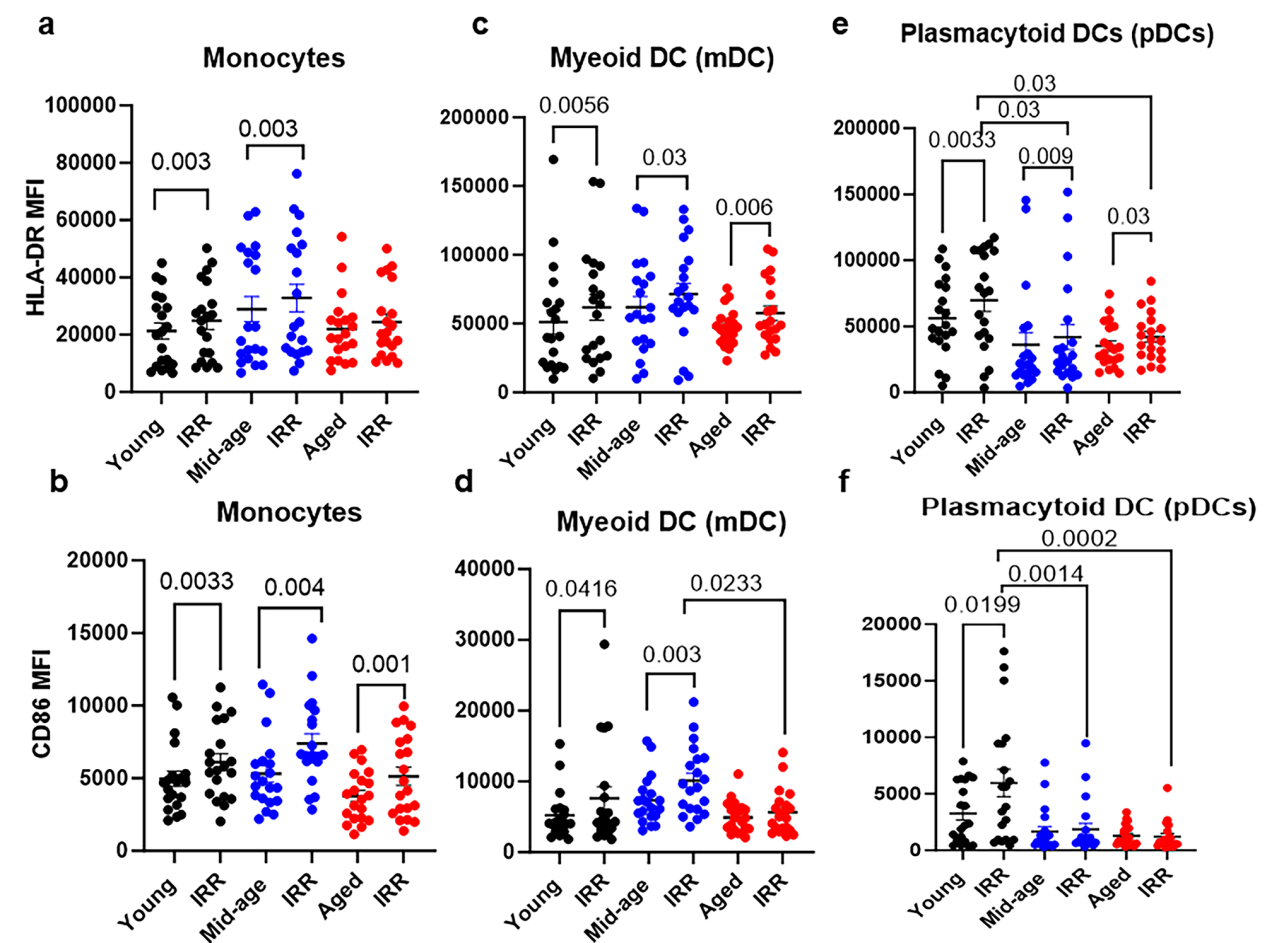
Activation of DCs and monocytes by SARS-CoV-2 in PBMCs as a function of age. PBMCs were stimulated with irradiated SARS-CoV-2 (IRR) virus for 24 h. The upregulation of HLA-DR and CD86 was determined using flow cytometry. **(a)** HLA-DR on gated CD14^+^ monocytes; **(b)** CD86 on gated monocytes; **(c)** HLA-DR on gated mDCs; **(d)** CD86 on gated mDCs; **(e)** HLA-DR on gated pDCs; **(f)** CD86 on gated pDCs. Mean + S.E. Young = 20; Mid-age-20; Aged = 20 subjects. The *P* value between unstimulated control and SARS-CoV-2-stimulated conditions in all age groups was calculated using a parametric paired t test; The *P* value comparing stimulated conditions of different aged groups was calculated using ordinary one way ANOVA followed by Tukey’s test

To investigate innate immune cell activation, we stimulated PBMCs from different age groups with irradiated SARS-CoV-2 virus for 24 h as described [30]. Since SARS-CoV-2 does not infect and multiply in DCs and monocytes, inactivated virus was used for stimulation [35–38]. We previously compared irradiated and heat-inactivated SARS-CoV-2 virus and demonstrated that irradiated virus is a better stimulator of the immune response [30]. Supernatants were collected and stored at -70 °C for cytokine and chemokine analysis. Cells were stained for DCs and monocytes using the gating strategy described previously and supplementary figure S2 [30]. The results indicate that HLA-DR is significantly upregulated on monocytes in young and middle-aged subjects upon stimulation with virus, but no significant upregulation was observed in aged subjects (Fig. 1a). When we determined the levels of the costimulatory molecule CD86 in monocytes, we found that in contrast to HLA-DR, CD86 was significantly upregulated in all age groups (Fig. 1b). In myeloid DCs (mDCs), HLA-DR was significantly upregulated in all age groups (Fig. 1c). However, CD86 was significantly upregulated only in young and middle-aged subjects but not in aged subjects (Fig. 1d). In fact, middle-aged subjects displayed significantly higher expression of CD86 upon stimulation than aged subjects (Fig. 1d). Similar to mDCs, HLA-DR was significantly upregulated on plasmacytoid DCs (pDCs) in all three age groups in stimulated versus unstimulated cells. However, in young subjects, HLA-DR expression was higher on stimulated pDCs than on stimulated middle-aged and aged pDCs (Fig. 1e). Compared to HLA-DR, CD86 showed even more of a defect in pDCs with age, with no induction of CD86 in middle-aged and aged pDCs upon stimulation (Fig. 1f). CD86 was only significantly upregulated on pDCs from young subjects (Fig. 1f). Taken together, these data suggest that aged DCs and monocytes are less responsive than middle-aged and young subjects to SARS-CoV-2. Among monocytes and DCs, pDCs displayed the maximum differences with age.

### Differential secretion of soluble mediators from SARS-CoV-2-stimulated PBMCs from various age groups at 24 h post stimulation with irradiated SARS-CoV-2

**Fig. 2:**
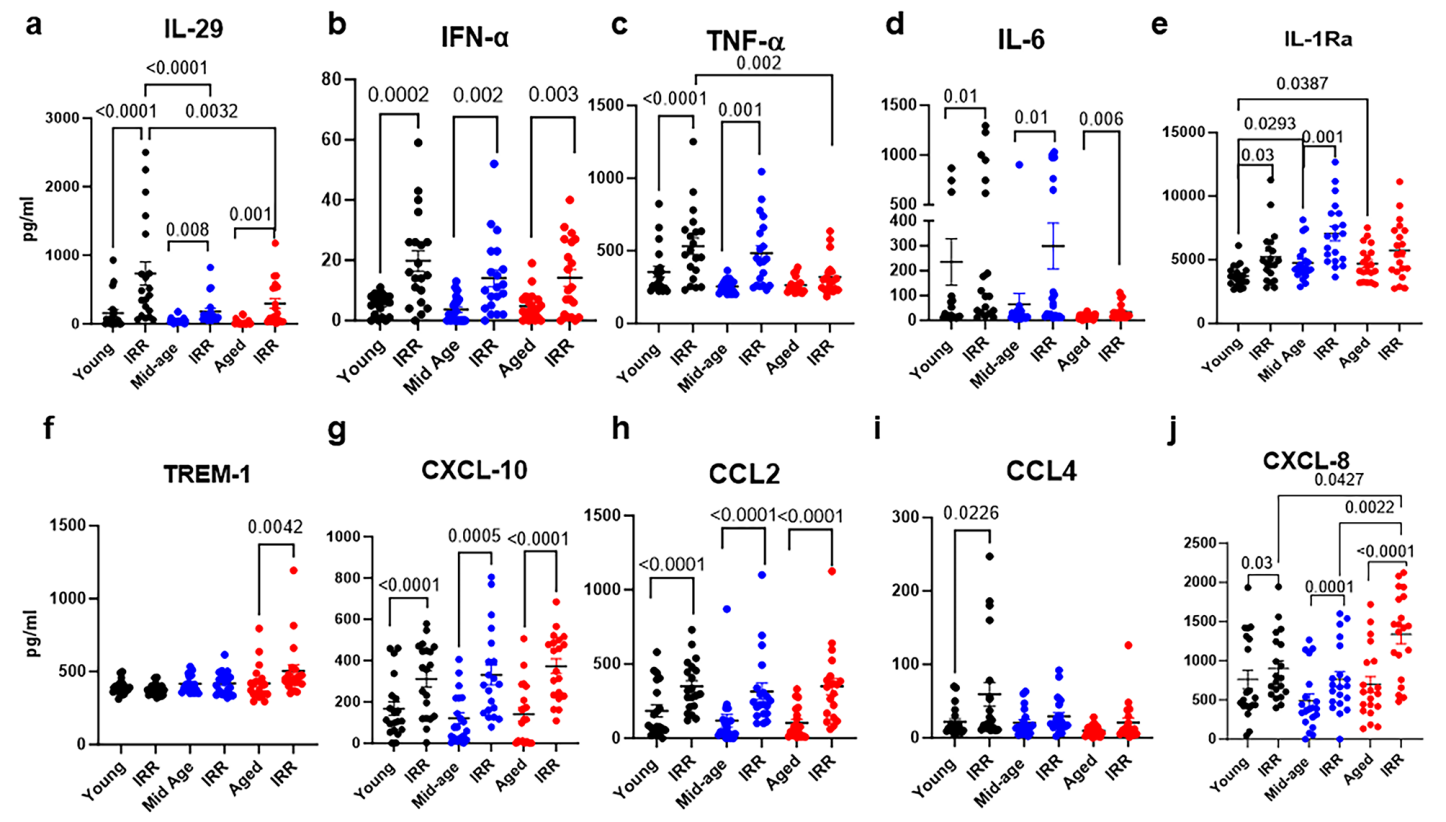
Differential secretion of soluble mediators from SARS-CoV-2-stimulated PBMCs 24 h post stimulation. PBMCs were stimulated with irradiated SARS-CoV-2 (IRR) for 24 h. Graphs depict the quantitation of cytokines/chemokines in the supernatant by multiplex. **(a)** IL-29; **(b)** IFN-α; **(c)** TNF-α; **(d)** IL-6; **(e)** IL-1Ra; **(f)** TREM-1; **(g)** CXCL-10; **(h)** CCL2; **(i)** CCL4; **(j)** CXCL-8. Mean + S.E. Young = 20; Mid-age-20; Aged = 20 subjects. The *P* value between the control and SARS-CoV-2-stimulated conditions in the three age groups was calculated using the paired t test (parametric). Significance between different age groups was calculated using ordinary one way ANOVA followed by Tukey’s test

Cytokines and chemokines were measured 24 h after stimulation with virus to determine innate immune responses. Some striking age-related differences were observed in antiviral cytokines. Type I and III interferons displayed interesting changes. IL-29 (type III IFN) was significantly upregulated in all age groups upon stimulation with virus. However, the mean secretion was 735pg/ml in young stimulated PBMCs while stimulated middle-aged PBMCs had a mean secretion level of 184 pg/ml and aged PBMCs 299 pg/ml (Fig. 2a, supplementary table ST5) so the middle-aged displayed 4-fold lower secretion and aged subjects displayed 2.5-fold lower secretion than young stimulated PBMCs. IFN-α (type I IFN) was secreted at significantly higher levels by all age groups upon stimulation. (Fig. 2b). Stimulation with virus led to the production of significant levels of TNF-α in young and middle-aged subjects but not in aged PBMCs (Fig. 2c). IL-6 was induced upon stimulation in all age groups. Young and middle-aged virus-stimulated PBMCs displayed a trend towards higher IL-6 secretion than in aged PBMCs but the differences were not significant (Fig. 2d). IL-1Ra was also induced significantly in young and middle-aged subjects but not in aged subjects upon stimulation with the virus (Fig. 2e). However, IL-1Ra levels were significantly higher at baseline in both aged and middle-aged subjects relative to young subjects (Fig. 2e). TREM-1, an inflammatory mediator, was significantly induced only in aged subjects, while middle-aged and young subjects did not show any change (Fig. 2f). Among the chemokines, CXCL-10 and CCL2 were significantly induced in all age groups (Fig. 2g & h), while CCL4 was significantly induced only in young subjects (Fig. 2i). CXCL-8 was also significantly induced in all three age groups upon stimulation. However, the levels were significantly higher in aged PBMCs than in middle-aged and young PBMCs (Fig. 2j). Taken together, antiviral responses such as IL-29 displayed a decrease as a function of increasing age, with aged subjects showing maximal defects in the secretion of TNF-α. In contrast to antiviral responses, inflammatory responses such as TREM-1 and CXCL-8 were higher on stimulation while IL-1Ra at baseline in aged subjects.

### Differences in cytokines and CD8 T-cell responses on day 7 post stimulation

We further investigated the adaptive immune CD8 T-cell responses to irradiated SARS-CoV-2 virus by determining cytotoxic T lymphocyte (CTL) induction and production of cytokines.

**Fig. 3:**
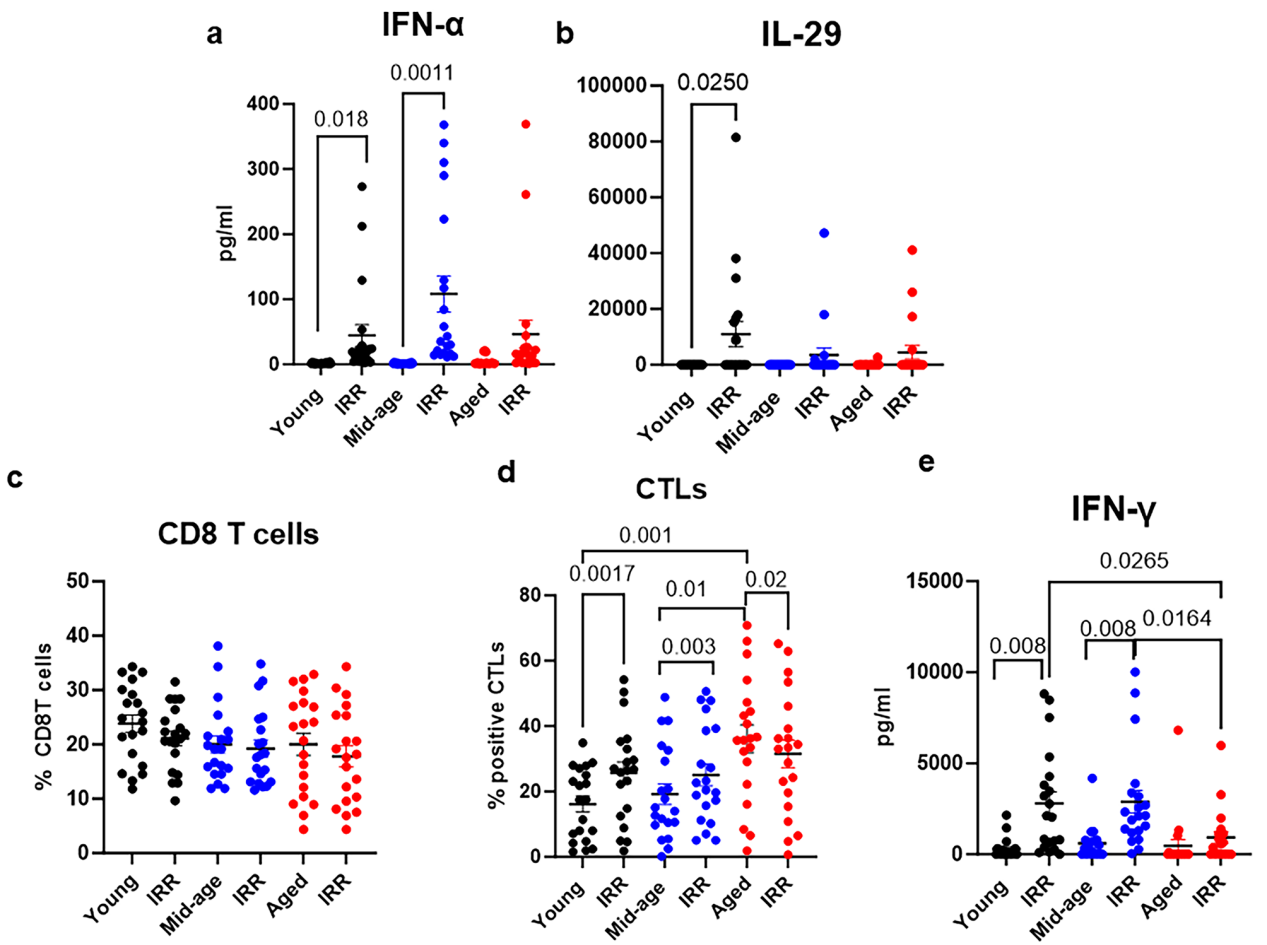
Differences in cytokines and CD8 T-cell responses on day 7 post stimulation. PBMCs stimulated with irradiated SARS-CoV-2 (IRR) were cultured for 7 days. The collected cells were stained for CD8, and granzyme B. The supernatant was quantitated for cytokines by multiplex. **(a)** IFN-α; **(b)** IL-29. Dot plot depicts the **(c)** % of viable CD8 T cells; **(d)** % of CTLs obtained using flow cytometry. **(e)** IFN-γ. Mean + S.E. Young = 20; Mid-age-20; Aged = 20 subjects. The *P* value between the control and SARS-CoV-2-stimulated conditions in the three age groups was calculated using the parametric paired t test. Significance between different age groups was calculated using ordinary one way ANOVA followed by Tukey’s test

Since we observed reduced levels of IL-29 and IFN-α in aged subjects at 24 h post stimulation, we determined the levels of these cytokines on day 7, as it was possible that the kinetics of production of these cytokines are slower in aged subjects. The levels of IFN-α continued to be higher in young and middle-aged subjects on day 7 post stimulation, while they returned to baseline in aged subjects (Fig. 3a). IL-29 levels continued to be significantly higher only in young subjects, with no visible induction in middle-aged and aged subjects (Fig. 3b).

CTL responses were in keeping with the cytokines. The percentage of granzyme B-positive CD8 T cells was significantly higher in PBMCs from aged subjects than in those from young and middle-aged subjects at baseline, even before stimulation. The percentage of CD8 T cells in all age groups were similar (Fig. 3c). However, upon stimulation with the virus, the percentage of CTLs displayed significant decrease in aged subjects. In contrast, there was significant increase in the induction of CTLs in young and middle-aged subjects (Fig. 3d). The reduced CD8 T-cell responses were also reflected in the cytokines. We also observed no significant increase in IFN-γ in the aged group, while it was significantly induced in young and middle-aged subjects  (Fig. 3e).

In summary, CD8 T-cell responses were impaired in aged subjects on stimulation but displayed increased activation at baseline in the absence of stimulation.

### Transcriptomic analysis of unstimulated PBMCs of different age groups

**Fig. 4:**
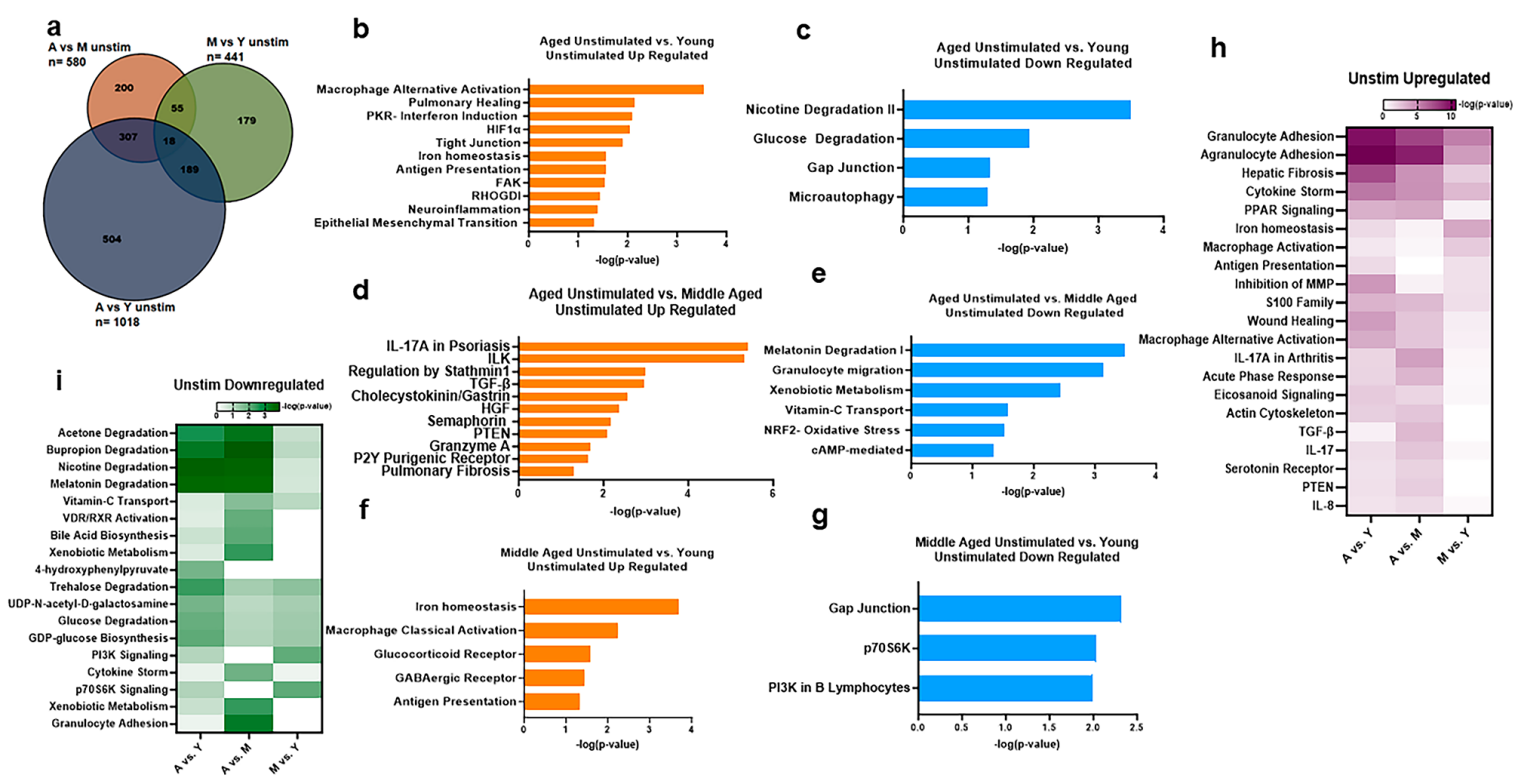
Transcriptomic analysis of unstimulated PBMCs of different age groups. We performed total RNA-seq analyses of unstimulated PBMCs from young (Y), middle-aged (M) and aged (A) individuals. **(a)** Venn diagram depicting the number of common and unique genes changed in A vs. Y, A vs. M and M vs. Y. Pathway analysis was done using Ingenuity pathway analysis (IPA) software. Bar graphs depicting selected up- and downregulated pathways for **(b)** A vs. Y upregulated; **(c)** A vs. Y downregulated; **(d)** A vs. M upregulated; **(e)** A vs. M downregulated; **(f)** M vs. Y upregulated; **(g)** M vs. Y downregulated. Heatmaps show selected **(h)** upregulated and **(i)** downregulated pathways after comparative pathway analysis of the three age groups. N = A-4; M-9; Y-4. Fisher’s Exact Test is used by IPA to calculate significance in pathway analysis including comparative pathway analysis. Values below 1.3 are not significant

To obtain a comprehensive picture of the changes induced with aging, we performed total RNA-seq on four samples (4 unstimulated and 4 stimulated) each from the aged and young subjects. For middle-aged individuals, we sequenced 9 samples (9 unstimulated and 9 stimulated). Details of the samples are provided in Supplementary Tables 1-ST1. First, we analyzed the RNA-sequencing data of unstimulated PBMCs from the three age groups to gain insight into the baseline/homeostatic age-related changes (Supplementary Table ST2A, ST2C, ST2E). Comparison of unstimulated gene changes between the three age groups revealed that aged versus young individuals showed maximum changes (Fig. 4a), with 1018 genes differentially expressed between the two groups. Of these, 504 were unique genes that were only changed in aged individuals when compared to young individuals and not when compared to middle-aged individuals (Fig. 4a). A total of 580 genes were differentially expressed in the aged group versus the middle-aged group (Fig. 4a), of which 200 were unique genes changed in the aged group relative to the middle-aged group. The middle-aged versus young group showed changes in only 441 genes, with 179 unique genes changed in the middle-aged group. Thus, aged, and middle-aged subjects showed differences in number of genes changed with respect to young.

We performed pathway analysis between unstimulated groups of different age groups (Supplementary Tables ST2B, ST2D & ST2F). Selected pathways are displayed in Fig. 4 (b-g). Pathway analysis of upregulated genes between aged and young subjects revealed significant changes in several pathways, including those related to macrophage alternative activation, wound healing, antigen presentation and HIF-1α (Fig. 4b). Interestingly, the PKR pathway for interferon induction was upregulated in aged subjects at baseline compared to young subjects (Fig. 4b). Pathway analysis of downregulated genes between unstimulated aged versus young subjects revealed changes in degradation pathways such as microautophagy (Fig. 4c). In contrast to aged versus young, aged versus middle-aged unstimulated upregulated genes displayed major changes in different pathways, including IL-17 A signaling, PTEN, Granzyme A, TGF-β and pulmonary fibrosis (Fig. 4d). When we performed pathway analysis of the downregulated genes between the same age groups, we observed downregulation of the NRF2 oxidative stress pathway, vitamin C transport, metabolism, and degradation-related pathways (Fig. 4e). Pathways that displayed changes in unstimulated/baseline middle-aged and young participants were partially similar to those in aged versus young participants. Iron homeostasis and antigen presentation pathways were upregulated in both middle-aged and aged individuals compared to young individuals, while macrophage classical activation and glucocorticoid receptor signaling were uniquely upregulated in middle-aged individuals (Fig. 4f). Downregulated pathways also showed a similar picture, with the gap junction pathway being downregulated in both middle-aged and aged subjects and PI3 kinase and p76S6 kinase uniquely downregulated in middle-aged subjects (Fig. 4g). Middle-aged subjects thus displayed more changes in metabolic pathways, while aged subjects had major changes in pathways associated with inflammation or resolution of inflammation.

We also performed comparative pathway analysis between unstimulated aged versus young, aged versus middle-aged and middle-aged versus young to obtain information about age-related changes in the common pathways as well as to identify pathways that were significantly up- or downregulated in a particular age group. Comparative pathway analysis enables visualization across multiple analyses with varying conditions such as age in our studies. We have shown a heat map of *p* value which is calculated using the fisher’s exact test. The higher the *p* value, more are number of genes that are changed in that pathway. Comparative pathway analysis of upregulated genes in the three age groups identified several pathways that displayed unique changes between age groups (Supplementary Table ST2G). Values equal to or above 1.3 are considered significant. Figure 4h displays selected pathways in a heatmap.

Pathways such as iron homeostasis, antigen presentation and inhibition of MMPs displayed changes from middle age onwards as they were significantly upregulated in both aged verses young and middle-aged verses young groups (Fig. 4h). Other pathways such as wound healing, phagosome formation, macrophage alternative activation as well as signaling pathways -PPAR, acute phase response, HIF-1α, Eicosanoid and actin cytoskeleton were significantly upregulated in both aged verses middle aged and aged verses young but not in middle aged verses young indicating that these pathways are upregulated on aging (Fig. 4h). In contrast other pathways such as IL-17, TGF-β, PTEN and IL-8 signaling were significantly upregulated only in the aged verses the middle-aged group and not in the aged verses young or middle-aged verses young group (Fig. 4h) suggesting that these pathways become upregulated later. There were also some pathways, such as macrophage classical activation and glutamate signaling/degradation, that were significantly upregulated uniquely in the middle-aged group versus the young group compared to the other two groups (Fig. 4h and Table ST2G). Pathways such as granulocyte, agranulocyte migration, fibrosis, and cytokine storm, were significantly upregulated in all three groups.

Comparative pathway analysis of downregulated genes of the three groups showed a different picture, with very few pathways displaying significant downregulation (Supplementary Table ST2G). Selected pathways are displayed in the heatmap (Fig. 4i). Degradation pathways such as acetone to methylglyoxal, melatonin, and nicotine were significantly downregulated in aged verses young and aged verses middle-aged groups but not in middle-aged verses young group (Fig. 4i). In contrast, vitamin C transport, VDR/RXR activation, and bile acid synthesis, and xenobiotic metabolic signaling were significantly downregulated only in aged verse middle-aged group and not the others suggesting that these changes occur after middle-age (Fig. 4i). Interestingly, pathways such as p70s6kinase and PI3kinase gap junction signaling were significantly downregulated only in the middle-aged versus the young group. (Fig. 4i). Trehalose and glucose degradation were downregulated in both aged and middle-aged groups when compared to young.

In summary, these data indicate that unique gene changes occur in aged and middle-aged groups compared to young groups that may influence their response to pathogens.

### Transcriptomic analysis in different age groups 24 h after stimulation with SARS-CoV-2

**Fig. 5:**
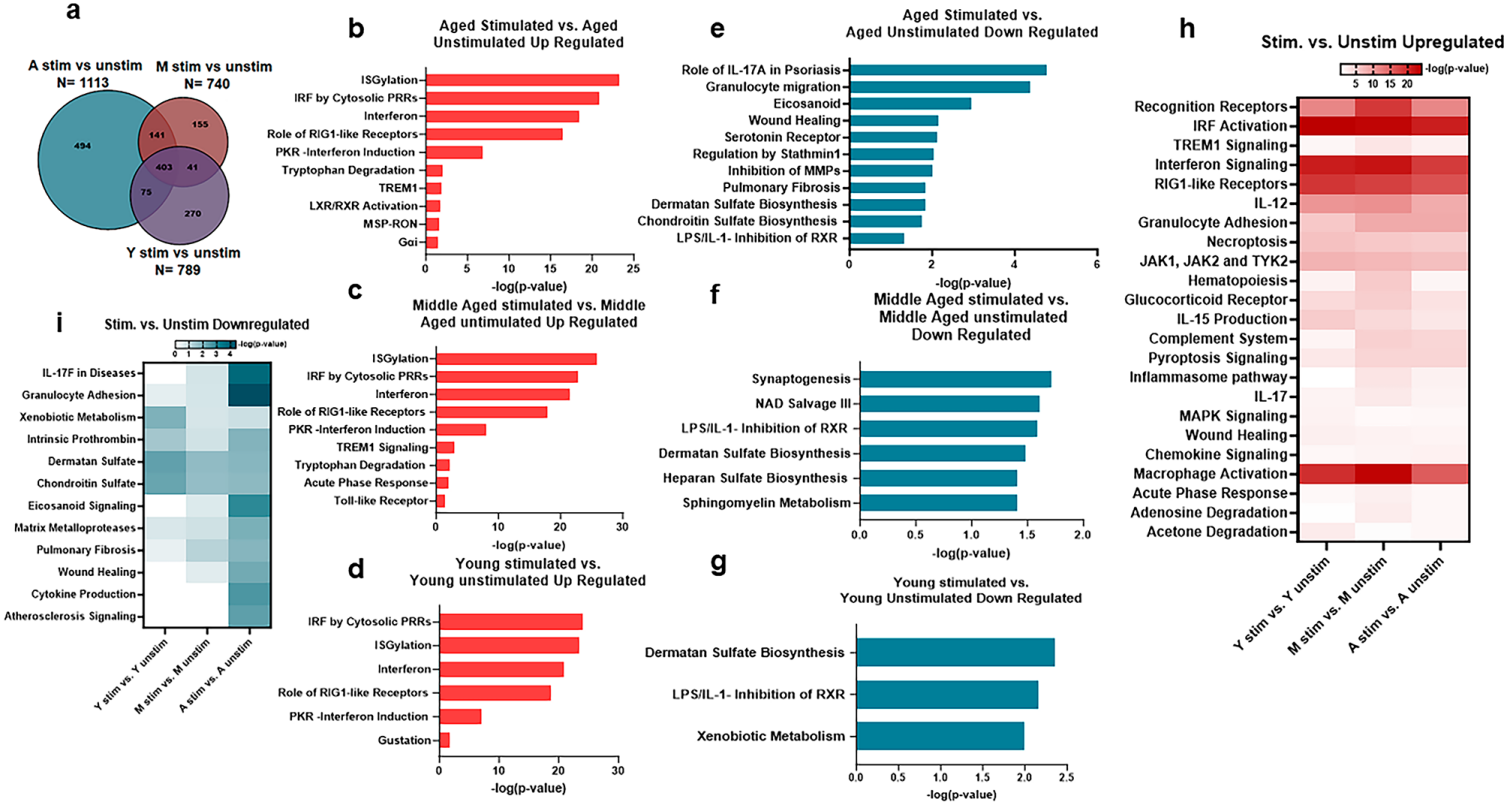
Transcriptomic analysis in different age groups 24 h after stimulation with SARS-CoV-2. We performed total RNA-seq analyses of unstimulated and SARS-CoV-2-stimulated (24 h) PBMCs from young (Y), middle-aged (M) and aged (A) individuals. **(a)** Venn diagram depicting the number of common and unique genes changed in A stimulated vs. A unstimulated, M stimulated vs. M unstimulated and Y stimulated vs. Y unstimulated. Pathway analysis was done using Ingenuity pathway analysis (IPA) software. Bar graphs depict selected up- and downregulated pathways for **(b)** A stimulated vs. A unstimulated upregulated; **(c)** M stimulated vs. M unstimulated upregulated; **(d)** Y stimulated vs. Y unstimulated upregulated; **(e)** A stimulated vs. A unstimulated downregulated; **(f)** M stimulated vs. M unstimulated downregulated; **(g)** Y stimulated vs. Y unstimulated downregulated. Heatmaps show selected **(h)** upregulated and **(i)** downregulated pathways after comparative pathway analysis of the three age groups. N = A-4 stimulated and 4 unstimulated; M-9 stimulated and 9 unstimulated; Y-4 stimulated and 4 unstimulated. Fisher’s Exact Test is used by IPA to calculate significance in pathway analysis including comparative pathway analysis. Values below 1.3 are not significant

To determine SARS-CoV-2 stimulation-induced gene changes in the three age groups, we analyzed the RNA-sequencing data between aged-stimulated versus aged-unstimulated, middle-aged stimulated versus unstimulated and young stimulated versus unstimulated groups (Supplementary Tables ST3A, ST3C, ST3E). In the aged samples, a total of 1113 genes displayed significant changes upon stimulation with SARS-CoV-2. Of these, 494 were unique genes only changed in this group (Fig. 5a). Middle-aged subjects displayed changes in 740 genes upon stimulation with SARS-CoV-2, with 155 unique genes. In contrast, in the young, 789 genes were changed, with 270 unique gene changes (Fig. 5a). Next, we performed pathway analysis of upregulated genes (Supplementary Tables ST3B, ST3C, ST3F). Selected pathways are displayed in the bar graphs (Fig. 5b-i). The results indicate that most of the significantly upregulated pathways were similar in all three age groups. These included pathways involved in antiviral responses (Fig. 5b, c, d). Some pathways, such as Toll-like receptor signaling, were upregulated only in the middle-aged subjects. In middle-aged and aged individuals, other pathways related to inflammation were upregulated, including the TREM-1 signaling pathway.

When we analyzed the downregulated pathways upon stimulation with SARS-CoV-2 (Supplementary Tables ST3B, ST3C, ST3F), we observed highly significant differences between the three age groups (Fig. 5e, f, g). Except for two, most of the pathways downregulated were different between the age groups. The aged subjects displayed the maximum number of downregulated pathways (Fig. 5e). Most of the pathways downregulated in aged subjects were related to IL-17 signaling, wound healing, eicosanoid signaling, etc., which are associated with host defense. In contrast, in middle-aged individuals, the pathways were related to NAD salvage and sphingomyelin metabolism (Fig. 5f). The NAD^+^ salvage pathway is essential for DNA repair. NAD^+^ also acts as a cosubstrate for a wide variety of enzymes, including PARPs, sirtuins, CD38/CD157 and SARM1, impacting metabolism, genomic stability, gene expression, inflammation, circadian rhythm, and stress resistance [39]. Accumulation of sphingomyelin is associated with tumor initiation and immune evasion [40]. Young subjects displayed downregulation of xenobiotic metabolism pathways (Fig. 5g).

Next, we performed comparative pathway analysis of upregulated genes of stimulated versus unstimulated conditions for all three age groups to determine the age-related changes in upregulated pathways (Supplementary Table ST3G). Selected pathways are displayed in the heatmap (Fig. 5h). Pathways of the coronavirus replication, inflammasome system, TREM-1 and chemokine signaling were significant upregulated on stimulation in both middle aged and aged groups but not in the young individuals (Fig. 5h). All these pathways are associated with severe COVID. Pathways that were significantly upregulated only in aged subjects were similar to the ones observed in Fig. 5b including Gαi and MSP-RON (Fig. 5h). Furthermore, several pathways displayed significant upregulation only in middle-aged subjects compared to both young and aged subjects. These included IL-33 signaling, acute phase response, and adenosine nucleoside degradation. Some host defense pathways such MAPK signaling in influenza pathogenesis, gustation and acetone degradation to methylglyoxal were significantly upregulated only in young (Fig. 5h). IL-10 signaling pathway that controls inflammation was upregulated in both middle-aged and young subjects but not the young.

Comparative pathway analysis was also performed for downregulated genes of the stimulated versus unstimulated groups for all ages (Supplementary table ST3G). Selected pathways are displayed in the heatmap (Fig. 5i). It showed much fewer changes than the upregulated comparison. Several pathways, including granulocyte diapedesis, IL-17 A pathways, eicosanoid signaling, pulmonary fibrosis, cytokine storm, and MMP inhibition, were significantly downregulated in only the aged subjects compared to young, middle-aged, and young subjects (Fig. 5i). NAD biosynthesis, salvage and sphingomyelin metabolism pathways displayed significant downregulation only in the middle-aged subjects. Pathways related to xenobiotic metabolism and coagulation were downregulated significantly only in the young subjects (Fig. 5i).

Altogether, the RNA-seq data indicate that antiviral responses decline while inflammatory responses increase with age.

## Discussion

SARS-CoV-2 infection is more severe and prevalent as we age [41–43]. The hospitalization and mortality rates are higher in elderly individuals despite vaccination [9, 10]. Several studies have investigated the underlying immune mechanisms, including innate immune mechanisms [44, 45]. However, information regarding the early activation of the innate immune cells DCs and monocytes is still scarce. In this study, we determined the activation of innate immune responses, including the activation of DCs and monocytes, by SARS-CoV-2 in young, middle-aged, and aged subjects.

Our results indicate that although DCs and monocytes are activated by SARS-CoV-2 in all age groups, their levels of activation are different. Monocytes and mDCs were only partially activated upon stimulation in the aged subjects, while pDCs displayed significantly impaired activation with age, which is consistent with our own and other studies (Fig. 1) [19, 46, 47]. More importantly, the reduced activation of pDCs was apparent from middle age, indicating that these key cell types for antiviral defense display signs of impairment early on. The flow cytometry results were supported by the cytokine and gene expression data. The production of IL-29, a major antiviral cytokine for respiratory viruses, was impaired in both middle-aged and aged subjects compared to young subjects. IL-29 or type III IFN function in a similar manner as type I IFNs except that their receptor is expressed primarily on mucosal epithelial surfaces. It provides protection without inducing damaging inflammatory responses. We previously reported impaired IL-29 secretion from DCs during aging [18, 19, 48]. IFN-α levels returned to baseline by day seven in the aged subjects, while they were still significantly upregulated in the middle-aged and young subjects. Other studies have also observed reduced IFN-α production in aged subjects with COVID-19 infection [45, 49]. Our data indicate that both pDC function and IL-29 production start displaying impairment from middle age. Thus, interventions aiming to improve these functions in aged subjects should start at an earlier age.

In contrast to antiviral defense cytokines, the production of inflammatory mediators, including TREM-1 and CXCL-8, was increased with age (Fig. 2). Both are associated with severe COVID-19 and were found to be upregulated in cytokine storms [50–53]. CXCL-8 attracts neutrophils to the site of inflammation and was found to be elevated in both mild and patients with severe COVID-19 and increased with disease progression [51]. We also observed upregulation of the chemokine signaling pathway with age. The role of TREM-1 in viral-associated complications, including pneumonia, is increasingly gaining prominence [52–55]. TREM-1 is a pattern recognition receptor that amplifies inflammatory responses by inducing the release of proinflammatory cytokines and chemokines, including CXCL-8. Soluble TREM-1 also blocks the production of anti-inflammatory cytokines such as IL-10. Comparative pathway analysis showed that IL-10 signaling pathway was downregulated in aged subjects. Interestingly, the TREM-1 signaling pathway was upregulated in both middle-aged and aged subjects upon SARS-CoV-2 stimulation. IL-1Ra was upregulated in young and middle-aged subjects upon stimulation. IL-1Ra is a receptor antagonist of IL-1 and therefore blocks the action of the cytokine controlling inflammation [56, 57]. Its upregulation in young and middle-aged individuals indicates the control of inflammation. In contrast, IL-1Ra was upregulated at the basal level in aged and middle-aged subjects, probably to control inflammation triggered by the IL-1/inflammasome.

The pathways downregulated upon stimulation in aging, including IL-17 signaling, inhibition of MMPs, serotonin receptor signaling and eicosanoid signaling, are all associated with severe COVID-19 and promote inflammation [58–63]. Higher IL-17 levels are associated with cytokine storm, pneumonia, and severe COVID-19 [60]. Eicosanoid signaling was also associated with severe COVID as Bronchoalveolar lavages (BALs) from severe COVID-19 patients displayed an abundance of inflammatory lipid mediators mostly eicosanoids such as prostaglandins and leukotrienes [63, 64]. Moreover, increased levels of eicosanoid prostaglandin D_2_ and phospholipase PLA_2_G2D were linked with poor outcomes in aged mice [65]. Another pathway downregulated in aged subjects was the inhibition of MMP signaling. Higher expression of MMPs such as MMP-2, 8 etc. were associated with tissue destruction in the lungs of severe COVID subjects [66]. The downregulation of these pathways with age may be a protective mechanism to control excessive inflammation in aging. In contrast to these pathways, serotonin receptor signaling that controls inflammation is downregulated in aged subjects. A recent study has shown a link between low serotonin levels and long COVID [67].

We also found some interesting changes with age at homeostasis that can affect COVID pathogenesis. Increased innate activation and inflammation were visible in aged subjects, as apparent by the upregulation of several pathways, including antigen presentation, wound healing, eicosanoid signaling, and inhibition of MMPs [64, 68]. All these pathways play a role in attracting inflammatory cells and aiding their movement. Pathways such as antigen presentation, inhibition of MMPs, and iron homeostasis were upregulated from middle age, again indicating that many changes are initiated early on. We have previously reported increased baseline activation of DCs in aged subjects as contributors to chronic inflammation [16, 69]. In addition, we observed increased PTEN signaling in aged subjects compared to that in middle-aged subjects. We also previously observed this and have shown that PTEN signaling is increased in aging and inhibits TLR responses [16]. Several pathways related to the degradation of sugars, melatonin, etc., that involve cytochrome P450 proteins were downregulated with age, which is in keeping with the age-associated decrease in drug metabolism reported in previous studies [70].

Iron homeostasis is an important immune regulator and is required for the activation of both innate and adaptive immune responses [71]. It can affect, among other things, macrophage polarization and T-cell activation and differentiation. The iron homeostasis pathway was significantly upregulated in both middle-aged and aged individuals relative to young individuals. However, pathways such as classical macrophage activation and glutamate receptor signaling were upregulated only in middle aged verses the young individuals. Both these pathways also enhance the immune responses [72, 73]. Pathways involved in metabolism, such as p70S6kinase and PI3kinase, were downregulated in middle-aged individuals only and not in aged individuals. Both of these proteins are involved in protein synthesis and are part of the mTOR pathway [74]. Downregulation of these pathways at homeostasis promotes macrophage activation [75]. It is well established that the PI3 kinase/mTOR pathway displays changes in elderly individuals [76]. However, we found that middle-aged subjects also display changes in this pathway, which may be responsible for the baseline activation of innate immunity.

The impaired innate immune responses with age also led to dysregulated CTL responses. In keeping with increased baseline innate immune activation and inflammation, CTL responses were higher at homeostasis in aged subjects than in both middle-aged and young subjects. This was accompanied by no significant increase in CTL activity upon stimulation with SARS-CoV-2. We have reported increased frequencies of granzyme B-positive CD8 T cells in aging [19]. Granzyme B also enhances inflammation and is involved in the pathogenesis of inflammatory diseases [77].

Most individuals in our study are vaccinated. Moreover, we do not have information about their COVID infection though they were not sick at the time of blood draw. Both these aspects may influence trained immunity. There are very few studies that have examined trained immunity in COVID-19. In most reports, the effect of trained immunity due to BCG, MMR or other vaccines is examined for protection against COVID-19. One study has investigated the effect of COVID-19 vaccination on trained immunity and the vaccine studied was chimpanzee adenovirus encoding the SARS-CoV-2 Spike glycoprotein (ChAdOx1 nCoV-19) [78]. Trained immunity was induced by the adenovirus and not the spike protein. In US, most subjects were vaccinated with either the Pfizer or Moderna vaccine. However, we do not have information about the type of vaccine used in our subjects. There are some reports of severe COVID 19 inducing trained immunity, but studies indicate that this does not enhance protection to subsequent infections and therefore its effect on immune cells is not clear [79].

## Conclusion

In summary, our study indicates that the response of DCs and monocytes, particularly pDCs, to SARS-CoV-2 stimulation is compromised with increasing age. There is decreased production of antiviral cytokines with a concomitant increase in inflammatory responses in both middle-aged and aged subjects. Both middle-aged and aged subjects also display increased baseline inflammation, although several inflammatory pathways in both age groups are different. The increased inflammation at homeostasis may compromise the response to pathogens, as there is increased baseline CTL activation in aging, which is impaired upon stimulation with SARS-CoV-2. This in vitro study can recapitulate several changes observed in aged subjects infected with SARS-CoV-2. Our study also indicates that several of these changes start at middle age and increase/decrease as a function of age. Interventions for preventing age-associated decline in immunity should therefore target middle-aged subjects. We also identified novel inflammatory pathways, such as TREM-1, which may be targeted in aging to reduce inflammation.

### Electronic supplementary material

Below is the link to the electronic supplementary material.

Supplementary Material 1

Supplementary Material 2

Supplementary Material 3

Supplementary Material 4

Supplementary Material 5

Supplementary Material 6

Supplementary Material 7

Supplementary Material 8

Supplementary Material 9

## Data Availability

The datasets used and/or analyzed during the current study are available from the corresponding author upon reasonable request. Raw data files for the figures 1-3 are in the supplementary table ST5. The sequencing files/data has been deposited in GEO (GSE254647).
