# Supplementary figures and images for: Changes in the innate immune response to SARS-CoV-2 with advancing age in humans
Source: Immun Ageing. 2024 Mar 21;21:21. doi: 10.1186/s12979-024-00426-3 (PMC10956333; doi:10.1186/s12979-024-00426-3)

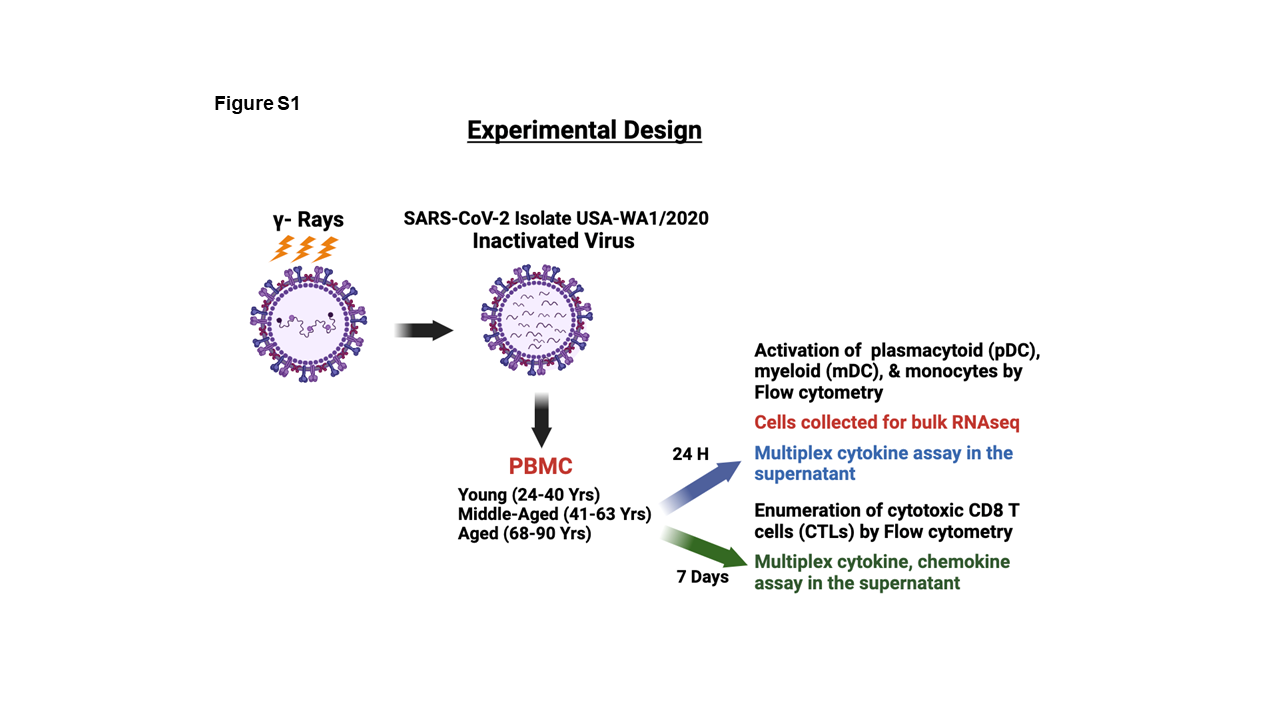

Supplement: Supplementary file 6 — Supplementary Material 6 [file 12979_2024_426_MOESM6_ESM.tif]

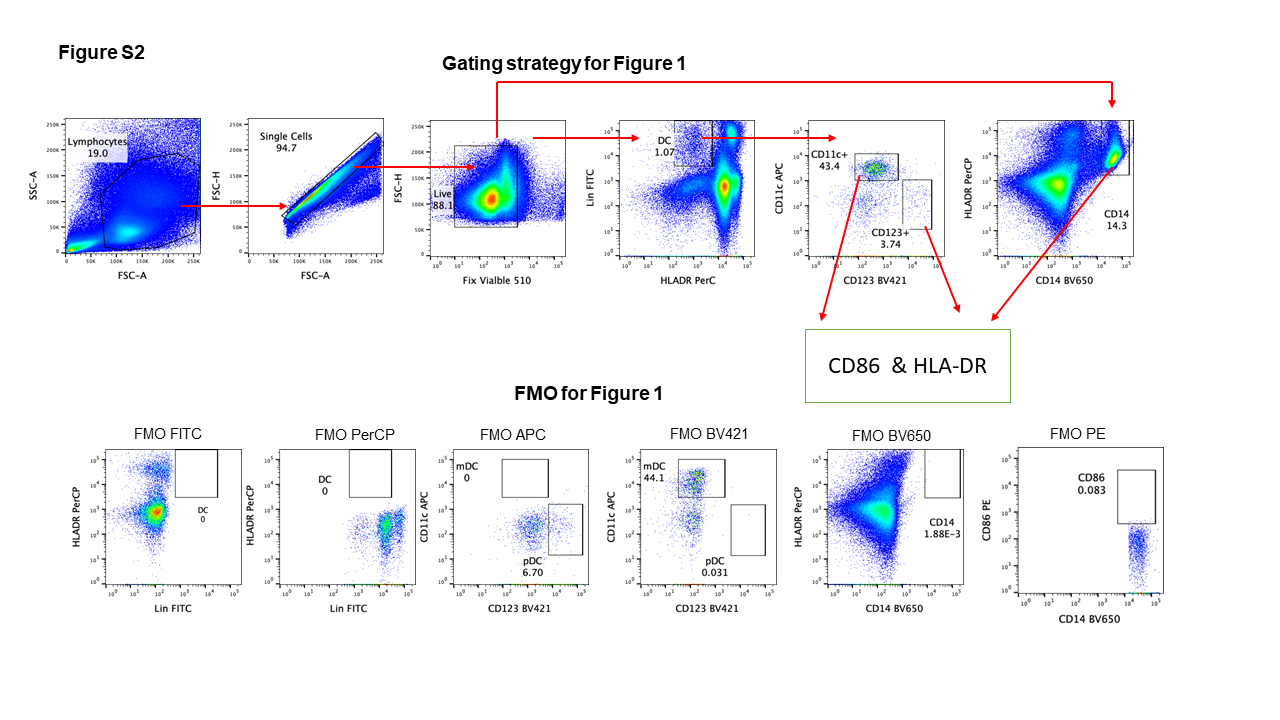

Supplement: Supplementary file 7 — Supplementary Material 7 [file 12979_2024_426_MOESM7_ESM.tif]

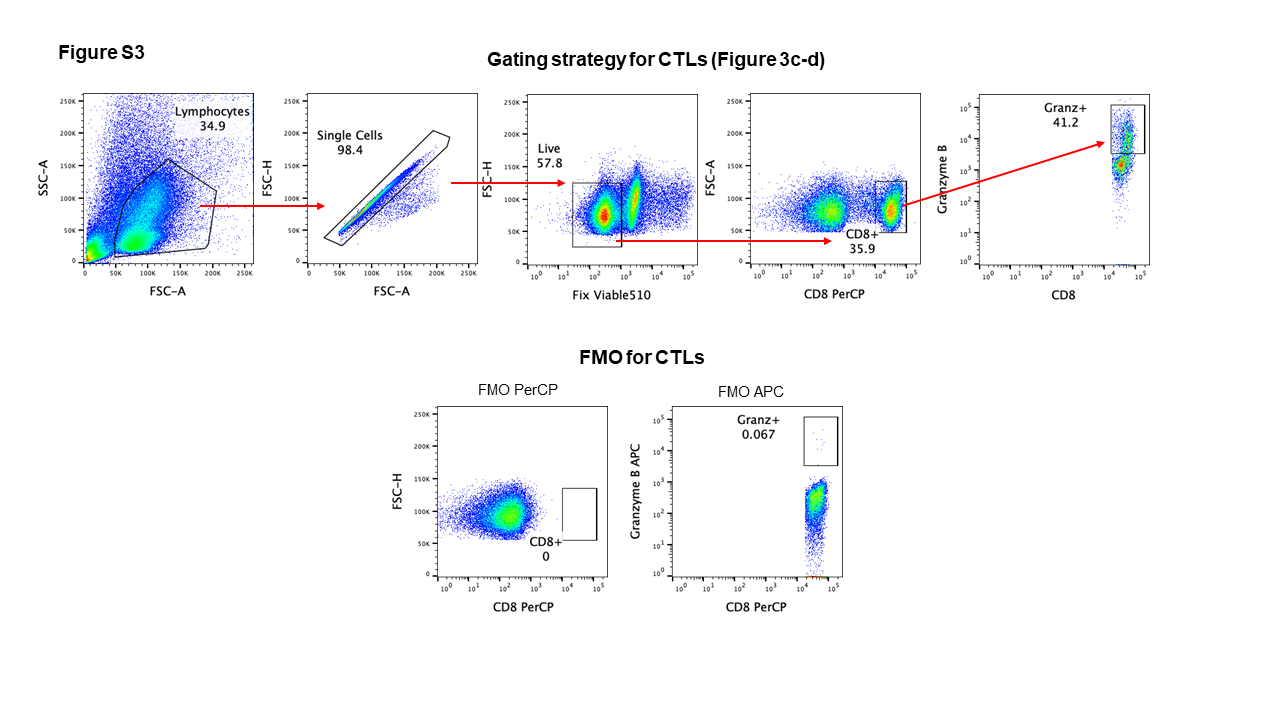

Supplement: Supplementary file 8 — Supplementary Material 8 [file 12979_2024_426_MOESM8_ESM.tif]
