# Supplementary material for: Changes in the innate immune response to SARS-CoV-2 with advancing age in humans
Source: Immun Ageing. 2024 Mar 21;21:21. doi: 10.1186/s12979-024-00426-3 (PMC10956333; doi:10.1186/s12979-024-00426-3)
Supplement: Supplementary file 9 — Supplementary Material 9 [file 12979_2024_426_MOESM9_ESM.docx]

**Supplementary materials description**

**FIGURES**

**Supplementary Figure S1:** Overview of the experimental design and methods used for each time point.

**Supplementary Figure S2:** Gating strategy and FMO for mDC, pDC and monocytes for figure 1.

**Supplementary Figure S3:** Gating strategy and FMO for CD8 and CTLs in figure 3c and 3d.

**TABLES**

**Supplementary Table ST1:** Patient characteristics and samples used for RNA-seq analysis.

**Supplementary Table ST2A:** 1018 differentially expressed genes in 4 unstimulated aged Vs 4 unstimulated young cases with corrected value p<0.05.

**Supplementary Table ST2B:** Canonical pathways of 1018 genes in 4 unstimulated aged Vs 4 unstimulated young cases with value p<0.05.

**Supplementary Table ST2C:** 580 differentially expressed genes in 4 unstimulated aged Vs 9 unstimulated middle-aged cases with corrected value p<0.05.

**Supplementary Table ST2D:** Canonical pathways of 580 genes in 4 unstimulated aged Vs 4 unstimulated young cases with value p<0.05.

**Supplementary Table ST2E:** 441 differentially expressed genes in 9 unstimulated middle-aged Vs 4 unstimulated young cases with corrected value p<0.05.

**Supplementary Table ST2F:** Canonical pathways of 441 genes in 9 unstimulated middle-aged Vs 4 unstimulated young cases with value p<0.05.

**Supplementary Table ST2G:** Comparative pathway analysis of 1018 genes in 4 unstimulated aged Vs 10 unstimulated middle-aged cases, 580 genes in 4 unstimulated aged Vs 4 unstimulated young cases, and 441 genes in 9 unstimulated middle-aged Vs 4 unstimulated young cases with value p<0.05.

**Supplementary Table ST3A:** 1113 differentially expressed genes in 4 stimulated aged Vs 4 unstimulated aged cases with corrected value p<0.05.

**Supplementary Table ST3B:** Canonical pathways of 1113 downregulated genes in 4 unstimulated aged Vs 4 stimulated aged cases with value p<0.05.

**Supplementary Table ST3C:** 740 differentially expressed genes in 9 stimulated middle-aged Vs 9 unstimulated middle-aged cases with corrected value p<0.05.

**Supplementary Table ST3D:** Canonical pathways of 740 downregulated genes in 9 unstimulated middle-aged Vs 10 stimulated middle-aged cases with value p<0.05.

**Supplementary Table ST3E:** 789 differentially expressed genes in 4 stimulated young Vs 4 unstimulated young cases with corrected value p<0.05.

**Supplementary Table ST3F:** Canonical pathways of 789 downregulated genes in 4 unstimulated young Vs 4 stimulated young cases with value p<0.05.

**Supplementary Table ST3G:** Comparative pathway analysis of 1113 genes in 4 unstimulated aged Vs 4 stimulated aged cases, 740 genes in 9 unstimulated middle-aged Vs 9 stimulated middle- aged cases, and 789 genes in 4 unstimulated young Vs 4 stimulated young cases with value p<0.05.

**Supplementary Table ST4:** Description of antibodies used for flow cytometry.

**Supplementary Table ST5:** Raw data values for figures 1-3 (each in a different sheet) with Mean and SEM for each column. The age and gender for each subject is provided in the first table- CD14 HLADR. Other tables are arranged to follow the same subject pattern.
